# Hepatitis B seroprevalence among HIV-positive adults in the Free State, South Africa

**DOI:** 10.4102/sajhivmed.v26i1.1654

**Published:** 2025-02-20

**Authors:** Devon Muir, Sabeehah Vawda, Phillip A. Bester, Cornel van Rooyen, Dominique Goedhals

**Affiliations:** 1 Division of Virology, Faculty of Health Sciences, University of the Free State, Bloemfontein, South Africa; 2 Division of Virology, National Health Laboratory Service, Bloemfontein, South Africa; 3 Division of Virology, PathCare, Pretoria, South Africa; 4 Department of Biostatistics, Faculty of Health Sciences, University of the Free State, Bloemfontein, South Africa

**Keywords:** hepatitis B virus, seroprevalence, HIV/HBV coinfection, South Africa, viral hepatitis

## Abstract

**Background:**

HIV and hepatitis B virus (HBV) coinfection impacts negatively on the prognosis for people living with HIV (PLHIV). HBV is relatively neglected compared to HIV in the South African setting, with limited coinfection seroprevalence data from the Free State.

**Objectives:**

This study aimed to determine the seroprevalence of current and previous HBV in PLHIV in the Free State, South Africa.

**Method:**

In this descriptive cross-sectional study, 1007 residual HIV viral load (VL) samples were selected and screened for markers of current HBV infection (hepatitis B surface antigen [HBsAg]) and previous exposure to HBV (total antibodies to hepatitis B core antigen [HBcTA]) between 01 February 2022 and 31 March 2022. Seroprevalence was assessed for municipal health districts, age, sex, and HIV VL categories.

**Results:**

HBsAg seroprevalence was 6.26% and HBcTA seroprevalence was 36.05%. HBsAg seroprevalence was significantly higher in men at 8.12% as compared to women at 4.38% (*P* = 0.014). HBcTA seropositivity was also higher in men than in women, at 42.97% for men and 29.08% for women (*P* < 0.0001). Peak HBsAg and HBcTA positivity was observed among the 40–59-year age group in both men and women. No significant difference was found in HBV seroprevalence and exposure between districts or HIV VL categories.

**Conclusion:**

These findings demonstrate that PLHIV in the Free State, South Africa, fall under the intermediate HBV prevalence category. HBV infection remains a public health concern and emphasis should be placed on identifying and addressing programmatic gaps regarding diagnosis and management.

**What this study adds:** This research contributes to South African HBV seroprevalence data in PLHIV, in a previously understudied population in the Free State province. Timely identification and treatment are necessary to achieve the 2030 World Health Organization HBV targets, alongside preventative measures, in this vulnerable population group.

## Introduction

According to the World Health Organization (WHO) Global Hepatitis Report, it is estimated that 254 million persons were chronically infected with hepatitis B virus (HBV) in 2022.^1^ HBV prevalence in Africa is estimated to be 5.8%.^1^ In HBV-endemic South Africa, approximately 3.5 million people are HBV infected, a conservative estimate, where the true case burden is likely much higher considering the irregularities of seroprevalence and census data.^2^ Global prevalence of HIV and HBV coinfection is estimated at 7.4%,^3^ although findings may vary depending on geographical region and HBV endemicity, with up to 25% of people living with HIV (PLHIV) co-infected in highly endemic areas.^4^ In South Africa, in PLHIV, the prevalence of HBV infection (positive hepatitis B surface antigen [HBsAg]) is estimated to be between 5% and 17%.^5,6,7,8^ HIV/HBV co-infected patients are more likely to have aggressive chronic HBV infection with higher viral titres, spontaneous seroreversions, occult infection, and liver-related mortality, thus surveillance and inclusion into HIV treatment and management programmes is necessary.^6,7,9,10,11,12,13,14,15^ HIV/HBV-coinfected patients are more likely to be men, and older than HIV mono-infected patients.^6,14^

Transmission of HBV occurs through routes commonly shared with HIV transmission, usually via contact with infected blood and bodily fluids. In highly endemic regions, with a lack of effective vaccination and preventative measures, the main route of transmission is ascribed to vertical transmission (perinatal infection), while in low endemic areas, horizontal transmission (in ages younger than 5) or sexual transmission routes (among adolescents and adults) are more common.^16,17^

Due to the risk of HBV chronicity in early childhood, vaccination was introduced into the South African Expanded Programme for Immunisation (EPI) in 1995, administered at 6, 10 and 14 weeks of age as a monovalent vaccine or hexavalent vaccine (DTaP-IPV-HIB-HepB), with a booster dose at 18 months of age.^16,18,19^ An HBV birth dose is currently not included in South Africa’s EPI vaccination schedule.^19^ Eliminating vertical transmission of HBV is critical to the WHO global hepatitis strategy, and requires a multipronged approach including implementation of universal screening programmes in pregnancy, re-allocation of health resources, antiviral prophylaxis/treatment, and a comprehensive vaccination series, including a birth dose.^20,21,22,23,24^ Owing to the fact that transmission occurs mostly before the age of 5 years, catch-up vaccinations for adolescents and adults are not yet feasible, considering that the adult susceptible population may be relatively small and resources could be better allocated for diagnosis and treatment in an endemic setting.^12,13,18^ The WHO has recommended catch-up HBV vaccination as an option for certain at-risk populations – unvaccinated adolescents in nonendemic settings, healthcare workers, unvaccinated household contacts of HBV-infected individuals, and those who would receive frequent blood products.^25^ The overall effect of vaccination is likely to be delayed in South Africa because of the relatively large proportion of chronically infected individuals, thus identifying high-risk individuals for intervention (including the HIV/HBV-coinfected population) will be a more cost-effective interim measure.^15,18,26^

HBV prevalence rates in South Africa are difficult to determine, with varying results.^7,16,18,27^ It was estimated in a systematic review conducted between 1965 and 2013, that South Africa had a HBV seroprevalence of 6.7%, while the 2016 Polaris Observatory Collaborators progression model estimated 7.2%.^2,15,16,28,29,30,31^ In urban areas, the prevalence can range from 1.3% (Soweto) to 7.4% (Durban), compared to rural areas with a prevalence as high as 15.5% (Eastern Cape) to 20% (Limpopo).^18,27,32,33^ Age prevalence estimates in South Africa estimate the peak for men between 30 and 44 years old, while for women the peak is between 30 and 34 years of age.^14,34^ These age peaks are likely a result of sustained transmission of HBV within the population, in the age groups that were born pre-vaccination implementation. Chronic carriage is higher in men, with different studies showing rates of between 5% (urban population) and 16% (rural population) in South Africa.^12,14,18,33,34,35^ In women, the seroprevalence is estimated to fall between 3% and 4% (urban areas) and 4% – 12% (rural areas).^14^ A household study in KwaZulu-Natal found that HIV/HBV-co-infected women of reproductive age seem disproportionately affected when compared to HBV mono-infected women, with seroprevalence rates above 6%.^8^ Higher HBsAg testing rates occur in women at an earlier age compared to men, likely because of higher rates of symptomatic infection and healthcare-seeking behaviour, such as antenatal and child-care visits.^34,36^

Population factors such as density (urban or rural), cultural practices, education level, socioeconomic status, sexual behaviour risk factors, and high-risk behaviour practices may influence transmission. A combination of these factors and access to healthcare services may impact subsequent identification of HBV in an HIV-positive adult population. It is thus expected that there will be regional and sub-regional variations in prevalence. In the Free State province, population densities vary widely, with the Xhariep district occupying the largest geographical area but accounting for only 5% of the provincial population, while the Mangaung metro occupies a relatively small geographic area in comparison yet accounts for 27% of the provincial population.^37^ These variances may have an impact on HBV seroprevalence at a district level in the Free State. Given the lack of local epidemiological data in the Free State, this study aimed to fill the need for region-specific data pertaining to HBV seroprevalence in an HIV/HBV-coinfected population.

## Research methods and design

### Study design

This was a descriptive cross-sectional study making use of residual HIV viral load (VL) samples from the Division of Virology, National Health Laboratory Service (NHLS) Universitas Academic Laboratories over the period of 01 February 2022 to 31 March 2022.

### Study population

Sample selection was stratified according to age, sex and municipal health district location. Sampling for male and female samples was approximately equal. This selective sampling method ensured that sampling was proportional between each Free State municipal health district, due to considerable geographic size and population variance among districts. Calculations for the required sample numbers per district were based on the last updated South African census data, while sample estimates for age were based on the 2021 population estimates published by the South African Department of Statistics.^38,39^ Samples were included if they met criteria for district, age, and sex, and were of sufficient volume. According to South African HIV management and treatment guidelines, only confirmed HIV-positive patients are referred for VL testing, thus by proxy all included samples were presumed HIV positive.^40^

Samples were stored at -80°C after collection and testing.

### Laboratory investigations

Serological testing made use of the DiaSorin Liaison XL Analyzer (Liaison XL, DiaSorin, Saluggia, Italy) in the laboratory, according to manufacturer’s instructions. To determine HBV seroprevalence, samples were screened for HBsAg using the Liaison XL Murex HBsAg Quant kit (DiaSorin, Saluggia, Italy), a direct two-step sandwich chemiluminescence immunoassay with a diagnostic sensitivity of 100% (95% confidence interval [CI]: 99.41% – 100%) and specificity of above 99.5% (95% CI: 99.89% – 100%). The manufacturer-reported assay range is from 0.030 to 150 IU/mL, where results below 0.05 IU/mL are considered non-reactive.^41^

Total antibodies to hepatitis B core antigen (HBcTA) were assessed to determine exposure to HBV (either current or previous infection), using the Liaison HBcTA kit (DiaSorin, Saluggia, Italy) which makes use of a two-step competitive chemiluminescence immunoassay format. This assay has a sensitivity of 100% (95% CI: 99.41% – 100%) and specificity of 98.72% (95% CI: 97.57% – 99.41%), according to the manufacturer. HBcTA values are expressed as an index, where an index value of less than 1 can be considered as positive.^42^ All reagents and consumables were stored as per the manufacturer’s instructions prior to testing.

Unexpected results included positive HBsAg and negative HBcTA results. Confirmatory testing of atypical HBsAg and HBcTA results was performed using a secondary electrochemiluminescence sandwich immunoassay, the Roche Elecsys HBsAg II on the Cobas e801 analyser (Roche Diagnostics, Mannheim, Germany), and retesting HBcTA using the Liaison HBcTA kit. If the confirmatory HBsAg remained positive while the HBcTA result was either negative or within equivocal range, these results were interpreted as possible current acute infection, where HBcTA seroconversion had not yet fully occurred. If the confirmatory HBsAg tested negative, the final HBsAg result for these specimens was regarded as negative.

### Interpretation of hepatitis B virus test results

In this study, HBV serostatus was defined as follows: (1) HBsAg positive: HBsAg positivity indicates current infection; (2) HBcTA positive: the presence of antibodies to HBV core antigen indicates exposure through natural infection (current or previous).

### Statistical analysis

The analysis was done by the Department of Biostatistics at the University of the Free State, using Statistical Analysis Software (version 9.4; SAS Institute Inc., Cary, North Carolina, United States). Age was categorised into three groups of 18–39 years, 40–59 years, and ≥ 60 years old for each district sample. HIV VL was clustered into three groups of lower than detectable limit (LDL), detectable between 50 copies/mL and 1000 copies/mL, and detectable at ≥ 1001 copies/mL. HBsAg seroprevalence and HBcTA seroprevalence were calculated for the sample population selected. Continuous variables were summarised by medians and percentiles, while categorical variables were summarised by frequencies and percentages. The Chi-squared (*χ*^2^) test was used to compare categorical variables. A *P*-value < 0.05 was considered statistically significant.

### Ethical considerations

An application for full ethical approval was made to the Health Sciences Research Ethics Committee of the University of the Free State, Bloemfontein, South Africa, and ethics consent was obtained on 10 February 2022 (HSREC approval number UFS-HSD2021/1870/2202). Individual informed consent was waived due to the use of de-identified residual diagnostic samples. The NHLS Academic Affairs and Research Management System (PR2225791) approved access to routine data required from the NHLS Central Data Warehouse (CDW) and use of de-identified residual diagnostic samples.

## Results

### Study population characteristics

A total of 1007 samples were included in the study. Table 1 summarises the basic study population characteristics. Stratification according to population size for sample selection per Free State municipal health district resulted in proportional sample sizes between districts with results as follows: Fezile Dabi 229 (22.74%), Lejweleputswa 226 (22.44%), Mangaung Metro 225 (22.34%), Thabo Mofutsanyana 227 (22.54%), and Xhariep 100 (9.93%). The median age in this study was 44 years old, while the minimum age was 18, and maximum 89. Sample sizes for each age grouping was as follows: category 1 (18–39 years old), 401 (39.82%); category 2 (40–59 years old), 403 (40.02%); and category 3 (≥ 60 years old), 203 (20.16%). Distribution of male to female samples was approximately equal, with 502 (49.85%) female samples and 505 (50.15%) male samples. The majority of patients, 634 (62.96%), had undetectable HIV VL, while 181 patients (17.97%) had a detectable HIV VL between 50 and 1000 copies/mL, and 192 (19.07%) patients had an HIV VL ≥ 1001 copies/mL.

**TABLE 1:** Study population characteristics (*N* = 1007).

Characteristic	*n*	%
**Municipal health district**		
Lejweleputswa	226	22.44
Mangaung Metro	225	22.34
Thabo Mofutsanyana	227	22.54
Fezile Dabi	229	22.74
Xhariep	100	9.93
**Age category (years)**		
18–39	401	39.82
40–59	403	40.02
≥ 60	203	20.16
**HIV VL category (copies/mL)**		
LDL	634	62.96
50–1000	181	17.97
≥ 1001	192	19.07
**Sex**		
Male	505	50.15
Female	502	49.85
**Sex by age category**		
Female (*N* = 502)		
18–39	200	39.84
40–59	202	40.24
≥ 60	100	19.92
Male (*N* = 505)		
18–39	201	39.80
40–59	201	39.80
≥ 60	103	20.40

VL, viral load; LDL, lower than detectable limit.

### Hepatitis B surface antigen and total antibodies to hepatitis B core antigen prevalence

Among the 1007 samples, 63 were HBsAg positive, with an overall HBsAg seroprevalence of 6.26%, and 363 samples were HBcTA positive, a seroprevalence of 36.05%. A small portion had equivocal HBcTA results (*n* = 23, 2.21%). The majority of the sampled population tested HBsAg negative (*n* = 944, 93.74%) and HBcTA negative (*n* = 621, 61.67%).

HBsAg prevalence varied between municipal health district, with the highest positivity found in Lejweleputswa (21/226, 9.29%) followed by Xhariep (7/100, 7%), Mangaung metro (13/225, 5.78%), Thabo Mofutsanyana (12/227, 5.29%) and Fezile Dabi (10/229, 4.37%). The calculated *χ*^2^ was 5.49, with *P* = 0.24, indicating that there was no statistically significant difference in HBsAg seroprevalence between the municipal districts. The Lejweleputswa health district had the highest HBcTA positivity (96/226, 42.48%), followed by Fezile Dabi (85/229, 37.12%), Mangaung metro (74/225, 32.89%), Thabo Mofutsanyana (76/227, 33.48%), and Xhariep (32/100, 32%) health district. No statistically significant difference was noted for HBcTA seroprevalence between municipal districts, with a calculated *χ*^2^ of 9.13 and *P* = 0.33. Table 2 summarises HBsAg and HBcTA seroprevalence results and association to the Free State municipal health districts.

**TABLE 2:** Hepatitis B surface antigen and total antibodies to hepatitis B core antigen seroprevalence and association with municipal health district (*N* = 1007).

Variable	HBsAg positive		HBsAg negative		*P*	HBcTA positive		HBcTA negative		*P*	HBcTA equivocal	
	*n*	%	*n*	%		*n*	%	*n*	%		*n*	%
**Municipal health district**	-	-	-	-	0.24	-	-	-	-	0.33	-	-
Lejweleputswa	21	9.29	219	95.63	-	96	42.48	124	54.87	-	6	2.65
Mangaung Metro	13	5.78	205	90.71	-	74	32.89	145	64.44	-	6	2.67
Thabo Mofutsanyana	12	5.29	212	94.22	-	76	33.48	145	63.88	-	6	2.64
Fezile Dabi	10	4.37	215	94.71	-	85	37.12	142	62.01	-	2	0.87
Xhariep	7	7.00	93	93.00	-	32	32.00	65	65.00	-	3	3.00

**Total**	**63**	**6.26**	**944**	**93.74**	**-**	**363**	**36.05**	**621**	**61.67**	**-**	**23**	**2.28**

HBsAg, hepatitis B surface antigen; HBcTA, total antibodies to hepatitis B core antigen.

HBsAg seroprevalence was significantly higher in men at 8.12% (41/505) as compared to women at 4.38% (22/502), with a calculated *χ*^2^ of 5.99 and *P* = 0.014. HBsAg positivity varied among age categories for both male and female participants, although a statistically significant difference was found only for the 18–39-year age group (*P* = 0.026). HBsAg seroprevalence in men was 7.46% in ages 18–39 years, 9.95% for ages 40–59 years, and 5.83% for ≥ 60 years. In women, positivity was consistently lower, at 2.55% in ages 18–39 years, 6.44% for ages 40–59 years, and 4% for ≥ 60 years. Table 3 summarises HBsAg seroprevalence findings with regard to sex and age.

**TABLE 3:** Hepatitis B surface antigen seroprevalence and association with age and sex.

By age category (years)	Male			Female			*P*
	*N*	HBsAg seroprevalence		*N*	HBsAg seroprevalence		
		*n*	%		*n*	%	
18 – 39	201	15	7.46	200	5	2.55	0.026*
40 – 59	201	20	9.95	202	13	6.44	0.190
≥ 60	103	6	5.83	100	4	4.00	0.550

**Total**	**505**	**41**	**8.12**	**502**	**22**	**4.38**	0.014*

HBsAg, hepatitis B surface antigen; *n*, number; *N*, total.

* , Significant finding.

HBcTA seropositivity was significantly higher in men (42.97%) than in women (29.08%), with a calculated *χ*^2^ of 23.55 and *P* < 0.0001. HBcTA positivity for sex and age was significantly higher among men than women in the 18–39-year (*P* = 0.0011) and 40–59-year age groups (*P* = 0.0015). The results demonstrate consistently lower HBV exposure for women across all age categories, although not significantly so in the ≥ 60-year age group. Table 4 summarises findings of HBcTA seroprevalence for sex and age.

**TABLE 4:** Total antibodies to hepatitis B core antigen seroprevalence and association with age and sex.

By age category (years)	Male			Female			*P*
	*N*	HBcTA seroprevalence		*N*	HBcTA seroprevalence		
		*n*	%		*n*	%	
18–39	201	76	37.81	200	48	24.00	0.0011*
40–59	201	100	49.75	202	65	32.18	0.0015*
≥ 60	103	41	39.81	100	33	33.00	0.2100

**Total**	**505**	**217**	**42.97**	**502**	**146**	**29.08**	< 0.0001*

HBcTA, total antibodies to hepatitis B core antigen; *n*, number; *N*, total.

* , Significant finding.

Table 5 summarises findings of HBsAg and HBcTA seroprevalence for age. Peak positivity occurred among the 40–59-year age group, of significance for HBcTA positivity with a calculated *χ*^2^ of 10.45, *P* = 0.03. There was no HBsAg seroprevalence difference among age category comparison overall (calculated *χ*^2^ of 4.28, *P* = 0.12).

**TABLE 5:** Hepatitis B surface antigen and total antibodies to hepatitis B core antigen seroprevalence and association with age.

Age category (years)	*n*	HBsAg positive		HBsAg negative		*P*	HBcTA positive		HBcTA negative		*P*	HBcTA equivocal	
		*n*	%	*n*	%		*n*	%	*n*	%		*n*	%
18–39	401	20	4.99	381	95.01	0.12	124	30.92	270	67.33	0.03*	7	1.75
40–59	403	33	8.19	370	91.81	-	165	40.94	227	56.33	-	11	2.73
≥ 60	203	10	4.93	193	95.07	-	74	36.45	124	61.08	-	5	2.46

**Total**	**1007**	**63**	**6.26**	**944**	**93.74**	**-**	**363**	**36.05**	**621**	**61.67**	**-**	**23**	**2.28**

HBsAg, hepatitis B surface antigen; HBcTA, total antibodies to hepatitis B core antigen.

* , Significant finding.

Table 6 summarises findings of HBsAg and HBcTA seroprevalence for HIV VL. Although not statistically significant (calculated *χ*^2^ of 1.64, *P* = 0.44), HBsAg positivity increased with detectable HIV VL at 5.52% for LDL, 7.18% for 50 copies/mL – 1000 copies/mL, and 7.81% ≥ 1001 copies/mL. No significant findings for differences in HBV exposure between HIV VL categories were noted (HBcTA calculated *χ*^2^ of 4.16, *P* = 0.38).

**TABLE 6:** Hepatitis B surface antigen and total antibodies to hepatitis B core antigen seroprevalence and association with HIV viral load.

Variable	*n*	HBsAg positive		HBsAg negative		*P*	HBcTA positive		HBcTA negative		*P*	HBcTA equivocal	
		*n*	%	*n*	%		*n*	%	*n*	%		*n*	%
**HIV VL (RNA copies/mL)**	-	-	-	-	-	0.44	-	-	-	-	0.38	-	-
LDL	634	35	5.52	599	94.48	-	229	36.12	389	61.36	-	16	2.52
50–1000	181	13	7.18	168	92.82	-	61	33.70	114	62.98	-	6	3.31
≥ 1001	192	15	7.81	177	92.19	-	73	38.02	118	61.46	-	1	0.52

**Total**	**1007**	**63**	**6.26**	**944**	**93.74**	**-**	**363**	**36.05**	**621**	**61.67**	**-**	**23**	**2.28**

HBsAg, hepatitis B surface antigen; HBcTA, total antibodies to hepatitis B core antigen; VL, viral load; LDL, lower than detectable limit.

Six cases of HBsAg-positive and HBcTA-equivocal (two results) or negative (four results) were identified in this sample group. These cases hailed from the Lejweleputswa (*n* = 3), Thabo Mofutsanyana (*n* = 2), and Xhariep (*n* = 1) districts. Four of the six cases occurred in men while the other two were women. All six cases had HBsAg values > 150 IU/mL on the Liaison XL Murex HBsAg Quant assay, and were confirmed positive on the Roche Elecsys HBsAg II assay.

## Discussion

Of the study sample of 1007 HIV-positive patients, comprising a combination of rural and urban populations, more than a third (36.05%) had evidence of HBV exposure and 6.26% had positive HBsAg results. The HBsAg prevalence observed in this group is comparable to Africa region estimates from the WHO Global Hepatitis Report (6.1%) and South African systematic review data between 1965 and 2013 (6.7%), although lower than the 2016 Polaris Observatory Collaborators report for the region (7.2%).^1,15,16,28,29,30^ A more recent study of prevalence of laboratory-confirmed HBV infection in South Africa found HBsAg positivity of 8.09% nationally in 2019.^34^ In comparison, the HBsAg positivity findings here were lower – possibly because of the nature of the population sampled. This HBsAg result may be an underestimation of true seroprevalence within the population, as functional cure may be intensified in an HIV/HBV-coinfected population because the majority would be on antiretroviral therapy (ART), with at least one active antiviral against HBV. A Zambian cohort study noted that HBsAg loss occurs in a higher proportion of HIV/HBV-coinfected patients relative to HBV mono-infected patients, likely due to recovery of HBV-specific immune control mechanisms in patients on HBV-active ART.^43^

HBV prevalence varied between municipal health districts in the Free State. Although not statistically significant, HBsAg seroprevalence and HBV exposure was higher in the Lejweleputswa district (9.29% and 42.48% respectively) when compared to others. Interestingly, Fezile Dabi had the second-highest HBV exposure rate (37.12%), but the lowest HBsAg positivity (4.37%). Varying prevalence rates could be due to transmission clusters occurring in certain populations (that may have been randomly sampled) within the health district, as well as the interplay of environmental (such as access to health facilities), cultural (such as behavioural practices), and host factors (such as sex), which may impact HBV exposure. Something to consider, which this study did not address, is differing genotype-specific seroclearance among different populations and communities in sub-Saharan Africa, which is predominately HBV genotype A, followed by D and E.^44^

HBsAg positivity among men was significantly higher overall than in women (8.12% vs 4.38%), and was consistently higher compared to women in each age category, although this only reached statistical significance in the 18–39-year age group. The seroprevalence difference between sexes is not unexpected and is in keeping with local and global literature findings showing higher HBsAg test positivity rate and chronic carriage in men across all age groups compared to women.^12,14,18,33,34,35,45,46^ It is known that immune and host responses differ between sexes – so-called sexual dimorphism – influenced also by environmental factors and external societal gender roles which may in turn influence health-seeking behaviours.^47^ These findings note a significant difference in HBV exposure (HBcTA seropositivity) between sexes, with rates consistently higher in men, peaking in the 40–59-year age group. Although not significant, overall HBsAg positivity and HBV exposure peaks in the 40–59-year age group, at 8.19% and 40.94%, respectively. In the South African HBV-endemic setting, these findings suggest that sustained transmission of HBV is ongoing within the population, especially in middle adulthood (and more so in men). Considering the age of this study population, only 8.72% (88 individuals) of the cohort would have been eligible for HBV vaccination, which was introduced into the South African EPI in 1995. It can thus be assumed that this preventative measure would have had little impact on the seropositivity of the majority of this cohort. HBV education and preventative measures in this age group should be emphasised to curb continuing transmission. It would be interesting to note HBV seroprevalence and exposure among a similar cohort in future, in order to contrast pre- and post-vaccine introduction variances.

HBsAg positivity increased slightly as HIV VL increased, from 5.52% in the LDL group, to 7.18% in the 50 copies/mL – 1000 copies/mL HIV VL group, and 7.81% in the ≥ 1001 copies/mL group. HBV exposure was more variable between HIV VL categories. Positive serological markers of HBV infection, namely positive hepatitis B core antibody status, has been associated with a delay in HIV VL suppression, and subsequent HIV VL rebound in HIV/HBV-coinfected patients on ART.^48^ The possibility of occult HBV infection (OBI) in this population group cannot be excluded, as it is known that approximately 12% of HIV/HBV-coinfected patients have OBI.^49^

Six cases of current HBV infection were identified where HBcTA was either negative or equivocal (HBsAg positive, HBcTA equivocal or negative). HBsAg positivity was confirmed using another assay to exclude false positivity, while the HBcTA result was retested using the same DiaSorin Liaison HBcTA assay, confirming the initial results. Permanent or intermittent loss of HBcTA has been noted in immunocompromised patients, including PLHIV, and HBcTA levels are generally lower in immunocompromised patients. A single HBcTA measurement cannot reliably exclude immunologically controlled inactive HBV infection in this group.^50^ Although a false-negative HBcTA result cannot be definitively excluded as no confirmatory assay was available, plausible explanations for these cases include either loss of HBcTA or early HBV infection, where antibody responses to HBcTA are not yet detectable or are in the process of seroconverting.

This study had both strengths and limitations. The primary strength is the measurement of HBV seroprevalence across a large study sample with approximately equal male to female ratios and age categories across the different municipal health districts. Inclusion of sex and age categories allowed for an understanding of groups at increased risk of HBV transmission and exposure, enabling identification of groups that the current screening and treatment programme does not adequately target. As a limitation, this study was not able to assess the impact of HBV immunisation, as child and adolescent age groups were not included in the sample. Due to financial constraints, it was not possible to include a full HBV testing panel, including hepatitis B surface antibody, hepatitis B core immunoglobulin M (IgM) and HBV VL. Additional testing would have enabled identification of immunity, acute or chronic cases, and occult HBV. Because of the single time point of sampling used for this study, we could not follow progress of HBV chronicity or reactivations and the course of HIV VL. As this population comprised only PLHIV, the results cannot be generalised to the rest of the South African population (those living both with and without HIV).

## Conclusion

This study demonstrates that PLHIV in South Africa fall into the intermediate HBV prevalence category. Men had higher HBsAg seroprevalence and HBV exposure across all age groups compared to women, peaking in the 40–59-year-olds in both sexes. Access gaps need to be identified within this group and improved engagement with healthcare services targeted in order to limit the risk of long-term complications. In this population, HBV prevalence increases with age and peaks in middle adulthood (age group 40–59 years old). Prevalence variation may occur within municipal health districts due to transmission clusters within community populations, but HBV transmission and exposure seem to be approximately equally spread across the Free State. It is important to ensure HBV infection is not neglected in PLHIV. PLHIV should be screened and appropriately managed for HBV infection at all healthcare visits to reduce transmission within the general population and limit morbidity and mortality within this high-risk group.
